# Suicide among national university graduate students in Japan from 2002 to 2021

**DOI:** 10.1002/pcn5.180

**Published:** 2024-03-08

**Authors:** Toshiyuki Marutani, Katsuhiro Yasumi, Kenji Saito, Takehiro Ibaraki, Jun‐ya Takayama

**Affiliations:** ^1^ Health Care Center Ochanomizu University Tokyo Japan; ^2^ Developmental Clinical Psychology, Graduate School of Humanities and Sciences Ochanomizu University Tokyo Japan; ^3^ Health Support Center Tokyo Institute of Technology Tokyo Japan; ^4^ Department of Mechanical Systems Engineering, Faculty of Engineering Shinshu University Nagano Japan

**Keywords:** graduate students, Japan, pandemic, risk, suicide

## Abstract

**Aim:**

Graduate students are exposed to various types of stress. Thus, they are prone to mental health problems, and the most devastating result is suicide. The aim of this paper is to reveal the status of suicide among graduate students in Japan for 20 years.

**Methods:**

We analyzed cumulative data on suicide among national university graduate students from annual surveys on causes of non‐graduation in Japan for the 2002–2003 through 2021–2022 academic years. We asked all national universities with graduate schools to complete the surveys, and the participation rate was 91.1%.

**Results:**

The total number of students in the surveys was 2,383,858, and the number of deaths by suicide was 347 (292 males, 55 females). Chi‐squared test results showed significantly higher suicide mortality rates for the following groups: male (*p* < 0.001), temporary leave (*p* < 0.001), repeating the same year (*p* = 0.006), master's level (*p* = 0.005), and majoring in engineering (*p* < 0.001). Psychiatric diagnoses were detected among 44 students (12.7%). The largest distribution (27 cases) of *International Classification of Diseases, Tenth Revision* (ICD‐10) codes among those whose diagnoses were evident was F3, mood disorders. Estimated motives for suicide were reported for only 36 students (10.4%), of which the most prevalent was job search failure. The most prevalent suicide method was hanging (151 cases, 43.5%).

**Conclusion:**

Our findings indicate that student support facilities should recognize higher‐risk groups for suicide among graduate students. Our study adds suggestions for suicide prevention on campus during future pandemics.

## INTRODUCTION

Graduate students are exposed to various types of stress and are thus prone to mental health problems.^1^ For example, a systematic review revealed that 34.8% of graduate students from 12 countries suffered from anxiety disorder.^2^ The American College Health Association National College Health Assessment found that graduate students have a slightly higher rate of suicidal ideation (12.7%) than undergraduates (12.0%), although rates of depression and anxiety among graduates are slightly lower than those among undergraduates (14.8% vs. 17.3% and 19.5% vs. 22.8%, respectively).^3^ Among their sample of graduate students, Garcia‐Williams et al.^4^ reported that 7.3% had suicidal thoughts, 9.9% had lifetime suicide attempt, 67.8% had mild to severe depression, and 95.4% felt nervous or worried a lot. Various types of abuses, hostile behavior by supervisors, depressive symptoms, and post‐traumatic symptoms were reported to augment graduate students’ suicidality.^5^, ^6^, ^7^

Reports on completed suicide cases among graduate students have been scarce. A study on suicides in 12 colleges in the United States for 10 years (1980–1990) showed that the suicide rate (per 100,000 students) among graduate students was 9.1, higher than each class year of undergraduates (1.7–5.4).^8^ More recently, in China, where the frequent suicides of graduate students have gained social attention, suicides during 2000–2019 (20 years) were reviewed by searching public information, such as media reports, websites, and articles. The most prevalent cause of suicide among the total of 150 cases was graduation pressure (31 cases, 20.7%), followed by depression (27 cases, 18.0%). The suicide rate could not be calculated.^9^

In Japan, where the top cause of death at ages 10–39 years in the general population remains persistently suicide, a 23‐year longitudinal survey on suicides among undergraduates (1989–2011) was reported.^10^ The Mental Health Committee of the Council of National University Health Administration Facilities in Japan launched an annual survey on non‐graduation causes (including death) among national university graduate students in 2002. The current study aimed to analyze 20 years of cumulative data (2002–2021, or 2002–2003 through to 2021–2022 academic years) on suicide among graduate students in Japan to contribute to suicide prevention on campus.

## METHODS

The Mental Health Committee of the Council of National University Health Administration Facilities has been conducting annual surveys on non‐graduation causes among national university graduate students in Japan since the 2002–2003 academic year.^11^, ^12^ This survey was designed as a complete enumeration, not a sample survey, and we asked all the national universities with graduate schools (98 in 2002, 87 in 2003 and 2004, 86 in 2005 through 2007 and in 2017 through 2021, and 85 in 2008 through 2016) to join the survey. The survey on death has been conducted as part of the survey on non‐graduation causes, collecting information on each student's educational level (master's or doctoral), major, student types (domestic or international), year of enrollment, gender, history of temporary leave, history of repeating the same year, age at death, cause of death, date of death, and information related to illness, accident, and suicide. Suicide‐related information included confirmed or suspected, location, method, diagnosis of psychiatric disease if applicable, *International Classification of Diseases, Tenth Revision* (ICD‐10) code, and other related information if collected from anyone concerned. The data were collected by student support division staff, academic affairs division staff, and/or health support division (health support center or similar name) faculty or staff of each university. The participation rate of the universities was 91.1%.

We calculated the suicide rates among the students of the following categories and performed chi‐squared tests: gender, master's/PhD level, educational level by gender, major, history of temporary leave, and history of repeating the same year. We then performed a residual analysis for educational level by gender, major, history of temporary leave by gender, and history of repeating the same year by gender. We used SPSS V. 25, and *p* values of <0.05 were considered significant. For the student type (domestic/international), we performed a Fisher's exact test using R Version 4.3.2. Up to two participating universities with a large number of students did not provide the subdivisions of student types each year, in which the number of all students was counted as that of domestic students. In terms of estimated causes of suicide, we manually analyzed related information and categorized the causes in 2002–2003 through to 2019–2020, and asked directly the causes in the 2020–2021 and 2021–2022 academic years during the coronavirus disease 2019 (COVID‐19) period, referring to the causal categories taken by the National Police Agency.^13^ In addition, estimated associations between suicide and the COVID‐19 pandemic were searched in the 2020–2021 and 2021–2022 academic years.^14^ This study was approved by the Ethics Review Committee of the Council of National University Health Administration Facilities.

## RESULTS

As shown in Table 1, the accumulated number of students for the 20 years was 2,383,858 (male, 1,716,590, female, 667,268). The accumulated suicidal cases of total, male, and female students were 347, 292, and 55, respectively. Average age at the time of suicide was 26.2 years (SD = 5.1); the median was 24.0, and the mode was 24.

**Table 1:** Suicide cases and rates per 100,000 students for academic years 2002–2003 to 2021–2022.

	No. of students	No. of suicide cases	Suicide rate^a^	*χ* ^2^	*df*	Adjusted residuals	*p*
Total	2,383,858	347	14.6				
*Gender*							
Male	1,716,590	292	17.0	25.380	1		<0.001
Female	667,268	55	8.2				
*Educational level*							
Master's	1,594,504	257	16.1	8.069	1		0.005
Doctoral	789,354	90	11.4				
*Educational level by gender*							
Master's level male	1,165,090	227	19.5	41.915	3	6.2	<0.001
Master's level female	429,414	30	7.0			−4.5	
Doctoral level male	552,010	65	11.8			−2.0	
Doctoral level female	237,344	25	10.5			−1.7	
*Major*							
Humanities	83,733	20	23.9	44.274	6	2.1	<0.001
Social science	172,912	22	12.7			−0.9	
Physical science	195,007	43	22.1			2.6	
Engineering	849,799	163	19.2			3.9	
Agricultural science	159,389	17	10.7			−1.5	
Medicine and health science	375,222	29	7.7			−4.1	
Education	188,101	13	6.9			−3.1	
*History of temporary leave*							
Yes	159,002	47	29.6	26.799	1		<0.001
No	2,224,856	298	13.4				
*History of temporary leave by gender*							
Yes, male	98,845	32	32.4	54.953	3	4.8	<0.001
Yes, female	60,157	15	24.9			2.2	
No, male	1,617,745	258	15.9			2.8	
No, female	607,111	40	6.6			−5.9	
*History of repeating the same year*							
Yes	262,631	54	20.6	7.562	1		0.006
No	2,121,227	291	13.7				
*History of repeating the same year by gender*							
Yes, male	171,435	41	23.9	33.982	3	3.4	<0.001
Yes, female	91,196	13	14.3			−0.1	
No, male	1,545,155	249	16.1			2.9	
No, female	576,072	42	7.3			−5.2	
*Domestic/international*							
Domestic	2,073,104	333	16.1	25.123	1		<0.001
International	310,754	14	4.5				
*Domestic/international by gender*							
Domestic male	1,544,571	280	18.1				<0.001^b^
Domestic female	528,533	53	10.0				
International male	172,019	12	6.9				
International female	138,735	2	1.4				

^a^ Per 100,000 students.

^b^ Fisher's exact test.

The suicide rate among male students was higher than that of female students (*p* < 0.001). By educational level by gender, male master's students had higher suicide rate than other groups (*χ* ^2^ = 41.915, *p* < 0.001, adjusted residuals 6.2 for male master's). Suicide rates per academic major showed significant differences between seven majors (*χ* ^2^ = 44.274, *p* < 0.001). A residual analysis indicated students majoring in engineering, physical science, or the humanities were at higher risk for suicide (adjusted residuals were 3.9, 2.6, and 2.1, respectively), while those majoring in medicine and health science, education, agricultural science, or social science were at lower risk (adjusted residuals were −4.1, −3.1, −1.5, and −0.9, respectively).

Students with a temporary leave or history of repeating the same year had higher suicide rates than those without (*p* < 0.001 and *p* = 0.006, respectively). In two cases, no information as to whether the student had taken a temporary leave was provided, and in another two cases, whether the student had a history of repeating the same year was unknown. In a residual analysis of history of temporary leave by gender, the female students without such history showed the lowest risk, whereas the male students without such history were at higher risk than the female students with such history (adjusted residuals: −5.9, 2.8, and 2.2, respectively). The same analysis of history of repeating the same year by gender showed that the female students without such history were at the lowest risk, whereas the male students without such history were at higher risk than the female students with such history (adjusted residuals: −5.2, 2.9, and −0.1, respectively; Table 1).

Even after considering the limitation of our data about domestic/international classification, the robust difference (*p* = 1.18e−11) assured a higher suicide rate among the domestic students than among the international students (Table 1).

Psychiatric diagnoses were reported for 44 students (12.7%). Twenty‐five students (7.2%) were recorded as having consulted a psychiatrist, but their diagnoses were unknown. The respondents confirmed that 94 students (27.1%) did not seek professional advice. The largest distribution (27 cases) of ICD‐10 codes among those whose diagnoses were evident was F3, mood disorders, among which 19 cases were depression, two cases were bipolar disorder, and six cases were unclear about the second digit of the code (Table 2).

**Table 2:** Psychiatric diseases among students who committed suicide.

		ICD‐10 code	No. of students	%
With psychiatric disorders	Diagnosis detected	F0	0	
		F1	0	
		F2	6	
		F3	27	
		F4	7	
		F5	0	
		F6	1	
		F8	2	
		F9	1	
		Subtotal	44	12.7
	Seeking psychiatric consultation, diagnosis unknown		25	7.2
	Total		69	19.9
Not seeking psychiatric consultation			94	27.1
Unknown			184	53.0

Abbreviations: F0, organic, including symptomatic, mental disorders; F1, mental and behavioral disorders due to psychoactive substance use; F2, schizophrenia, schizotypal and delusional disorders; F3, mood (affective) disorders; F4, neurotic, stress‐related and somatoform disorders; F5, behavioral syndromes associated with physiological disturbances and physical factors; F6, disorders of adult personality and behavior; F8, disorders of psychological development; F9, behavioral and emotional disorders with onset usually occurring in childhood and adolescence.

Estimated motives for suicide were reported for only 36 students (10.4%), of which the most prevalent was job search failure, followed by academic failure (Table 3). In the two academic years during the COVID‐19 pandemic, no cases were estimated to have a direct association with COVID‐19, and there were three and one cases of indirect association in the 2020–2021 and 2021–2022 academic years, respectively (Table 4). Among descriptions of related information in the survey form, upon which the categorization of estimated motives for suicide used herein is based, there was one international student case affected by social isolation in 2020 because the student suffered from physical problems and wanted his family to come to Japan, but COVID‐19‐related restrictions prevented them from flying to and entering Japan. Another international student case was also estimated to be affected by travel restrictions during the pandemic. He had been psychologically unstable such that his father had asked him to come home, but he did not, and probably could not, and he committed suicide. A Japanese case in 2020 had a job search problem related to COVID‐19.

**Table 3:** Estimated motives for suicide (multiple answers allowed) (*n* = 36).

	*n*	%
Academic failure	9	25.0
Career concerns	3	8.3
Job search failure	13	36.1
Personal relationships with friends/professors	0	0.0
Love relationship problems	5	13.9
Life hardship	0	0.0
Discord in parent–child relationship	2	5.6
Isolation/loneliness	3	8.3
Worry/effects of illness	2	5.6
Other	4	11.1

**Table 4:** Estimated association with COVID‐19.

	2020–2021		2021–2022	
	(*n* = 20)		(*n* = 22)	
	*n*	%	*n*	%
Direct association	0	0.0	0	0.0
Indirect association	3	15.0	1	4.5
No association	4	20.0	9	40.9
Unknown	13	65.0	12	54.5

Suicide methods, reported in 272 cases (78.4%), are shown in Table 5. The most prevalent was hanging (151 cases, 43.5%). One freezing case was conducted on a snowy mountain. Seven of eighteen oral poisoning cases involved overdosing on prescribed medicine, including suspected cases. Others included taking potassium cyanide and aconite. Fifteen of 36 gas poisoning cases used a kind of briquette called “Rentan” (a solid fuel formerly common in general households until around 1965, these days used mainly for heating and cooking in outdoor activities), producing carbon monoxide intoxication. Others included inhaling hydrogen sulfide and carbon dioxide intoxication by dry ice exposure. Suffocation included four cases of inhaling helium gas.

**Table 5:** Suicide methods.

	*n*	%
Hanging	151	43.5
Jumping	46	13.3
Gas poisoning	36	10.4
Oral poisoning	18	5.2
Suffocation	11	3.2
Electrocution	4	1.2
Cutting	3	0.9
Drowning	2	0.6
Freezing	1	0.3
Unknown	75	21.6

## DISCUSSION

Among the general population in Japan, the male suicide rate is higher than the female suicide rate.^15^ Our data on gender differences were in line with those of the general population. Males are at a higher risk of suicide globally as well.^16^ Regarding the gender differences among the students with histories of temporary leave and repeating the same year, our data suggest that gender difference is a factor that poses a higher risk of suicide than history of temporary leave or repeating the same year. We will discuss later the overall and gender‐related differences between the students with and those without these histories.

Regarding educational level, master's students, especially male master's students, seemed to be at higher risk. A review of suicide among graduate students in China reported that the number of suicide cases in master's programs was higher than that in doctoral programs (94 and 56 cases, respectively), but suicide rates were not calculated due to the lack of data on the number in the denominator.^9^ In the United States, a recent study showed that suicidal ideation was more problematic among doctoral than master's students, and an old 10‐year longitudinal study of suicide reported that students aged 25 years or over were at higher risk for suicide than younger students.^3^, ^8^ In our study, the median of the age at suicide was 24.0 years and the mode was 24, which suggested that younger students among graduate students had elevated risk for suicide. However, this does not suggest that doctoral students are in a safer environment than master's students. The Japanese government has increased the capacity of graduate schools since 1991, from 68,739 master's students and 29,911 doctoral students in 1991 to 162,458 and 75,295, respectively, in 2021.^17^ Academic posts did not follow this augmentation and, at present, a shortage of academic posts is prevalent globally. This causes job search difficulty following the completion of studies even for those students forced to neglect their personal lives due to the tremendous amount of research work.^2^, ^18^ Risks for suicide may await after doctoral studies.

In Japan, 12.4% of all undergraduates (national, prefectural, municipal, and private) entered a master's degree program in 2022, of whom 43.1% were physical science, 38.0% engineering, 26.5% agricultural science, 4.3% education, 4.2% humanities, 4.2% medicine and health science, and 2.5% social science students.^17^ Our results show that suicide risk was highest for engineering students, followed by those of physical science and humanities students. Joining a master's program was so prevalent among the physical science and engineering students that some students might not have been sufficiently prepared for the heavy workloads assigned by demanding professors. Others might not have secured the type of employment they had expected, even if it is generally believed that proceeding to the master's level would give students a higher probability of securing a job at a better company. The employment rate among graduate students of the humanities was the lowest among the majors that we considered throughout the study period. The employment rate of master's graduates of the humanities in 2022 was 46.5%; for engineering 87.7%, physical science 76.0%, agriculture 78.8%, social science 59.6%, education 65.6%, and medicine and health science 75.7%. The employment rate of PhD graduates of the humanities in 2022 was 40.7%; for engineering 71.6%, physical science 68.6%, agriculture 72.3%, social science 49.9%, education 66.3%, and medicine and health science 79.7%.^17^ In this study, estimated motives were detected among only 36 cases, of which 13 cases (36.1%) involved job search failure (Table 3). Taken together, the low employment rate could be a background factor in suicide among humanities students.

The students with history of temporary leave and those with history of repeating the same year had higher suicide rates than those without when we compared overall and within the same gender. These results are consistent with the second most common estimated motive: academic failure (Table 3). In Japan, repeating the same year or repetition (*Ryûnen* in Japanese) is applied from senior high school. This system of delaying grade promotion is not used before then even for students whose grades do not reach the levels required by the governmental curriculum guideline. In senior high school, students with low academic achievements can avoid repeating the same year as long as they meet the required number of attendance days. The *Ryûnen* rate among high school students was 0.3% in 2021.^19^ Thus, most Japanese university students are not familiar with *Ryûnen*, which may mentally damage more Japanese students than students in other countries, although no previous reports have been published on this issue.

International students tend to have mental health problems due to various problems, such as cultural differences and language barriers,^20^ and thus are at risk of suicide.^21^ However, our data suggest that domestic students are at higher risk of suicide than international students regardless of gender. To the best of our knowledge, no previous reports have compared suicide rates between domestic and international students. Unmet interpersonal needs were identified as a factor associated with suicidal ideation not only among international students, but also among domestic students.^22^, ^23^ Minami et al.^24^ reported that international students in Japan were resilient. Taken together, we speculate that many international graduate students in Japan could cope with various stress and at least manage to avoid catastrophes even if they have mental health problems.

Approximately 90% of those who die by suicide are estimated to have had a psychiatric disorder.^16^, ^25^, ^26^ In our data, 94 cases (27.1%) did not seek psychiatric consultation, which indicates that at least one out of four students did not seek medical advice to assist with their problems. Among the ICD‐10 codes recorded, the most prevalent was depression (19 cases), which is consistent with previous psychological autopsy studies saying that depression is the most common psychiatric disorder in suicide cases.^16^, ^25^, ^26^ However, alcohol dependence and substance abuse, the secondary common psychiatric disorder in suicidal cases, were not present in our data. There were only a few cases of developmental disorders, such as autism spectrum disorder (F8) and attention deficit hyperactivity disorder (ADHD, F9), whereas a self‐administered Internet survey in Japan revealed that university students with high levels of autistic traits and of ADHD traits were at higher risk of poor academic performance.^27^ A limitation of our study is that information on the existence of psychiatric diseases for 184 cases (53.0%) was unavailable.

The most prevalent estimated motive for suicide in 36 cases was job search failure. In *Nature*'s 2019 PhD survey, 56% of respondents reported that their first choice for a career was in academia although there is a global shortage of jobs at universities and colleges, as we mentioned above.^18^ In Japan, in addition to academic post shortages, major companies have been hesitating to hire PhD graduates.^28^ This problem is also related to career concerns. Isolation and loneliness are common reasons for suicide,^29^ and there was one international student case affected by social isolation under the COVID‐19 travel restrictions.

Our surveys also asked about estimated associations with COVID‐19 in the 2020–2022 academic years (Table 4). One indirect association case (other than the one we described above) was also an international student case related to travel restrictions caused by the pandemic. Our unpublished data show that the most prevalent cause of temporary leave among international students was travel restrictions in both 2020 and 2021 (*n* = 273, 32.1% of all the temporary leave cases of international students, and *n* = 361, 37.3%, respectively). The travel restrictions included immigration and emigration restrictions and visa issuance suspension as well as significant reductions in flights and accordingly unaffordable elevated flight fares or flight suspension. Deprivation of their freedom of movement devasted mental health among international students, which could lead to suicide.

Finally, the most prevalent form of suicide was hanging, a highly fatal method of suicide, both in Japan and many other countries.^30^, ^31^ Although only seven cases used prescribed medicine, overdosing on prescribed drugs was reported to be a common supplementary means of suicide.^32^ Shimane et al.^33^ reported that in Japan, the abuse of prescribed psychiatric medication was recognized as problematic and benzodiazepines were the most commonly overdosed drugs. A longitudinal nationwide survey on drug abuse among inpatients and outpatients in hospitals with psychiatric wards indicated that the over‐the‐counter (OTC) drugs use rate has been increasing obviously since 2018, especially among the younger population (35.3% for OTC drugs and 20.8% for sleeping medicines and/or anti‐anxiety medicines among patients in their twenties who used drugs within 1 year before the survey in 2022).^34^ Accordingly, many more cases might have overdosed on prescribed medicines, or OTC drugs during the most recent several years, before they elected a fatal approach.

To conclude, our findings indicate that student support facilities should recognize higher‐risk groups for suicide among graduate students, such as male gender, master's level, younger age, a history of temporary leave or repeating the same year, majoring in engineering, physical science, or the humanities, with mood disorders, a background with job search failure, and academic failure. In future pandemics, the isolation of international students caused by travel restrictions also should be addressed. Considering our study's limitation of small sample size for estimated motives for suicide, further investigation is needed for better suicide prevention measures on campus.

## AUTHOR CONTRIBUTIONS

*Conceptualization*: Toshiyuki Marutani. *Data curation*: Toshiyuki Marutani and Katsuhiro Yasumi. *Formal analysis*: Toshiyuki Marutani, Katsuhiro Yasumi, and Jun‐ya Takayama. *Investigation*: Toshiyuki Marutani, Katsuhiro Yasumi, and Kenji Saito. *Project administration*: Katsuhiro Yasumi. *Writing—original draft*: Toshiyuki Marutani. *Writing—review and editing*: Toshiyuki Marutani, Katsuhiro Yasumi, Takehiro Ibaraki, Kenji Saito, and Jun‐ya Takayama.

## CONFLICT OF INTEREST STATEMENT

The authors declare no conflict of interest.

## ETHICS APPROVAL STATEMENT

This study was approved by the Ethics Review Committee of the Council of National University Health Administration Facilities.

## PATIENT CONSENT STATEMENT

N/A

## CLINICAL TRIAL REGISTRATION

N/A

## Data Availability

The data are not publicly available due to their containing information that could compromise the privacy of each case.
